# Sudden Death of a 17‐Month‐Old Chimpanzee ( *Pan troglodytes* ) due to a Congenital Undiagnosed Ventricular Septal Defect

**DOI:** 10.1111/jmp.70070

**Published:** 2026-03-13

**Authors:** Ivanela Kondova, Merel Langelaar, Arno A. W. Roest, Peter J. Heidt

**Affiliations:** ^1^ Biomedical Primate Research Centre Rijswijk the Netherlands; ^2^ Willem‐Alexander Children's Hospital of the Leiden University Medical Centre Leiden the Netherlands

**Keywords:** congenital heart defect, heart failure, sudden death

## Abstract

Ventricular septal defect (VSD) is a congenital defect that is frequently observed in humans. We report the sudden death of an infant chimpanzee due to a congenital undiagnosed VSD. During necropsy, the examination of the heart revealed a VSD, dilatation of the left ventricle and myocardial hypertrophy of the right ventricle.

## 1 Introduction

Chimpanzees ( *Pan troglodytes* ) are genetically and physiologically close to humans, and documentation of naturally occurring congenital anomalies is rare. In humans, congenital heart defects (CHDs) are the most frequent congenital abnormalities at live birth. Structural abnormalities of the heart present at birth are well documented in human medicine but reports of such conditions in non‐human primates, and especially great apes, are rare. Ventricular septal defect (VSD) is one of the most commonly observed congenital conditions in human newborns, resulting in abnormal communication between the left and right ventricles. It allows oxygen‐rich blood to move back into the lungs instead of being pumped to the rest of the body. The oxygen‐rich blood mixes with oxygen‐poor blood. These changes may increase blood pressure in the lungs and require the heart to work harder to pump the blood around. Small VSDs cause no problems and usually close on their own. Babies with medium or larger VSD may need surgery early in life to prevent complications.

This case report is selected from the archives of the Biomedical Primate Research Centre (BPRC) in the Netherlands. The research on chimpanzees at the BPRC ended in 2003, and in 2006, all the chimpanzees were donated to different zooparks in Europe.

We describe a sudden death due to a VSD with a diameter of ±1 cm in a young chimpanzee from the BPRC colony in the Year 2002. This report aims to contribute to the limited literature on CHDs in great apes and support comparative pathology and clinical management in zoological settings.

## 2 Case Report

A 17‐month‐old male chimpanzee was found dead in his group‐cage on 10‐06‐2002. The animal was in good body condition with unremarkable clinical history. In an attempt to reanimate the animal, heart massage was performed by the veterinarian without result. Necropsy was performed immediately after the death of the animal.

A multiplicity of subcutaneous haemorrhages was observed during the necropsy. However, the quantity of the haemorrhages was not considered to be related to the death of the animal, but considered to be related to the reanimation attempts. Histopathological examination verified the acute character of the haemorrhages.

**FIGURE 1:**
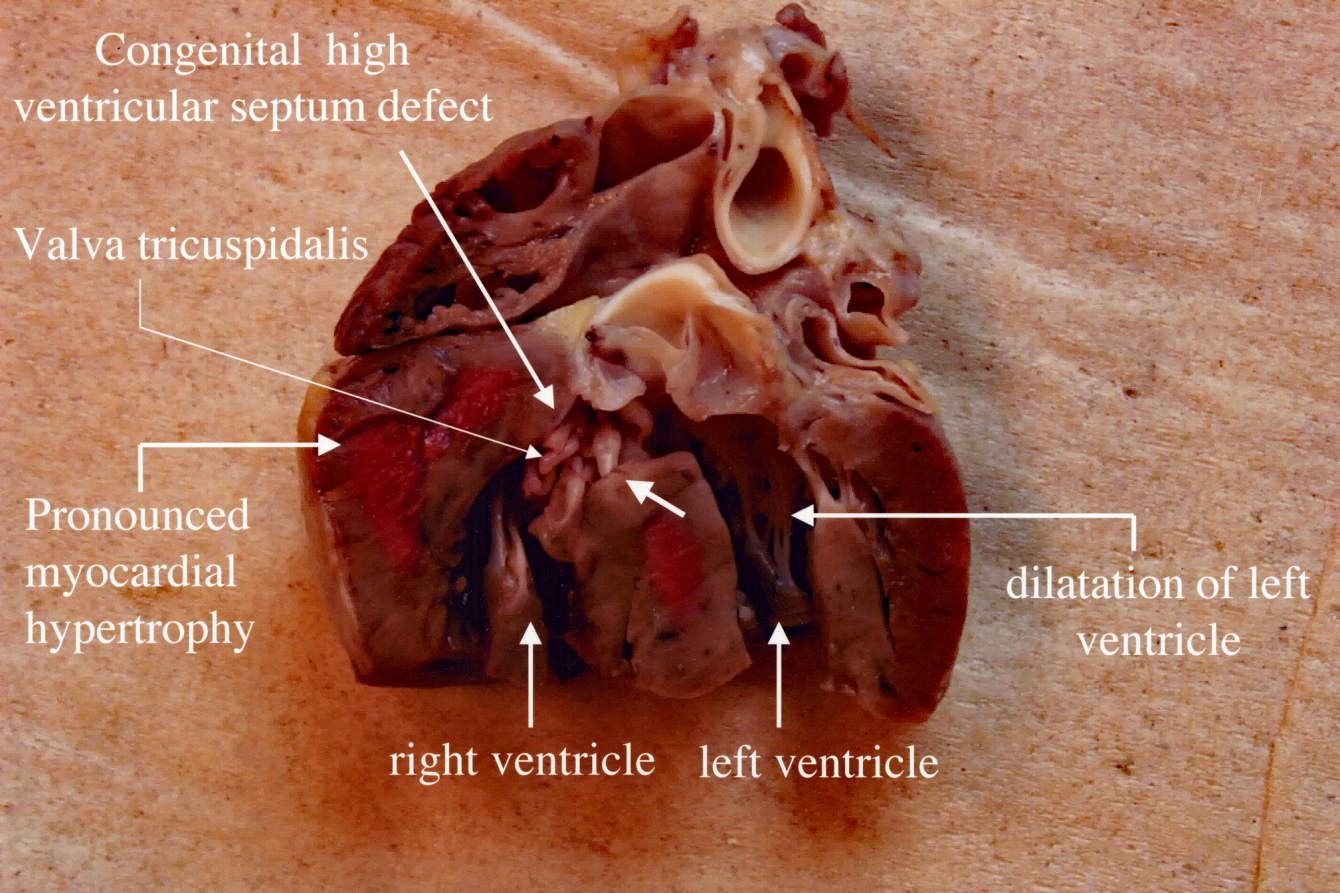
Gross appearance of the heart showing the ventricular septal defect.

The necropsy showed that the death of the animal was caused by a high‐positioned VSD of ±1 cm diameter, leading to failure of the circulatory system (Figure 1). Furthermore, dilatation of the left ventricle and myocardial hypertrophy of the right ventricle were evident. No other lesions that could be related to heart failure were found during the necropsy.

**FIGURE 2:**
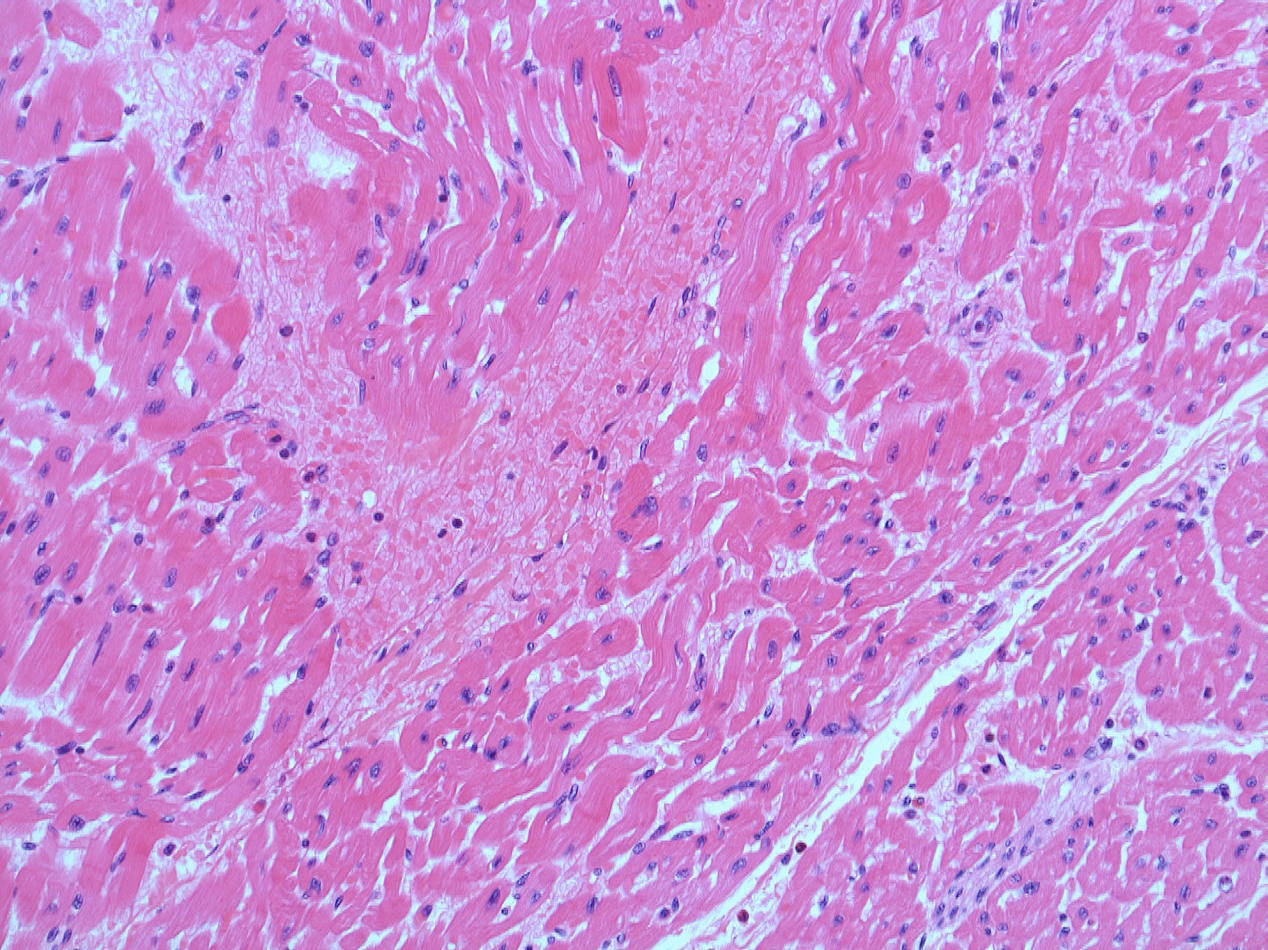
Microscopic findings of the heart (H&E staining, ×200). Segmental fibre‐degeneration with reactive resorptive inflammatory infiltrates and intramural extravasated erythrocytes (haemorrhage).

Histological examination revealed multifocal segmental fibre degenerations with reactive resorptive inflammatory cell infiltration accompanied by multifocal intramural haemorrhages in the myocardium (Figure 2).

## 3 Discussion

A congenital VSD in a chimpanzee is mainly observed during necropsy. In the case that we present, the infant chimpanzee showed no signs indicating a VSD, such as poor eating, failure to thrive, fast breathing or breathlessness and easy tiring. The finding of a hypertrophied right ventricle and the sudden death without any clinical signs suggest that pulmonary hypertension due to increased pulmonary flow through the VSD played an important role in this unexpected death.

We came across only one published case of a VSD in a chimpanzee. This heart abnormality was found during necropsy in a prematurely born chimpanzee in which after birth heart murmur, tachypnoea, dyspnoea and disturbances of blood flow were observed [1]. Cases of a VSD in other non‐human primates are a case in a Bonnet macaque ( *Macaca radiata* ) [2], a case in a 15‐year‐old Sumatran orangutan ( *Pongo abelii* ) [3] and a case in a 3‐year‐old gorilla ( *Gorilla gorilla gorilla* ) [4]. A VSD was also observed in other animal species: pika [5], alpaca [6], horse [7] and calves [8]. The incidence in different animal species is unknown. However, a congenital VSD is regularly observed in human children because of the healthcare in developed countries, but it can exist undiagnosed, leading to the sudden death of a child [9]. The rarity of sudden death in affected children was demonstrated in a multicentre study from 10 countries, in which only two cases (1%) of lethal VSD were found out of a total of 186 cases of sudden death [10]. In a population‐based cohort study, including more than 25 000 newborns born in the greater Copenhagen area, a total of 850 newborns (3.3% of 25.556) with a VSD were identified. The newborns underwent echocardiography within 60 days after birth. After 1 year, 83.5% of all VSDs had closed spontaneously, resulting in a decrease of prevalence from 3.3% at birth to 0.5% in 1‐year‐old children [11].

Since the incidence of congenital VSDs in human primates is around 3% it can be expected that this percentage is also occurring in non‐human primates, for example chimpanzees. The numbers of congenital VSDs observed in non‐human primates in the literature are low. This may be due to the fact that they are often not noticed. They are mostly reported in case studies rather than large‐scale prevalence studies, which is the reason that the correct percentages are missing. It is unlikely that the incidence in non‐human primates is different from that in humans. The chance of a young chimpanzee dying from this defect, based on the human percentages, is therefore small. That we diagnosed such a case in the BPRC chimpanzee colony with a limited number of births (67 births between 1976 and 2000) was therefore unexpected, based on the fact that only in 0.5% of 1‐year‐old human children this defect is (still) present.

## Conflicts of Interest

The authors declare no conflicts of interest.

## Data Availability

The data that support the findings of this study are available from the corresponding author upon reasonable request.
